# Intuitionistic Fuzzy Weighted Linear Regression Model with Fuzzy Entropy under Linear Restrictions

**DOI:** 10.1155/2014/358439

**Published:** 2014-10-29

**Authors:** Gaurav Kumar, Rakesh Kumar Bajaj

**Affiliations:** ^1^Singhania University, Pacheri Bari, Jhunjhunu, Rajasthan 333515, India; ^2^Jaypee University of Information Technology, Waknaghat 173234, India

## Abstract

In fuzzy set theory, it is well known that a triangular fuzzy number can be uniquely determined through its position and entropies. In the present communication, we extend this concept on triangular intuitionistic fuzzy number for its one-to-one correspondence with its position and entropies. Using the concept of fuzzy entropy the estimators of the intuitionistic fuzzy regression coefficients have been estimated in the unrestricted regression model. An intuitionistic fuzzy weighted linear regression (IFWLR) model with some restrictions in the form of prior information has been considered. Further, the estimators of regression coefficients have been obtained with the help of fuzzy entropy for the restricted/unrestricted IFWLR model by assigning some weights in the distance function.

## 1. Introduction

In statistical analysis, regression is used to explore the relationship between *k* input variables **x**_1_, **x**_2_,…, **x**_*k*_ (also known as* independent variables* or* explanatory variables*) and the output variable **y** (also called* dependent variable* or* response variable*) from *n* sets of observations. In linear regression, the method of least-squares is applied to find the regression coefficients *β*_*j*_, *j* = 0, 1,…, *k*, which describe the contribution of the corresponding independent variable **x**_*j*_ in explaining the dependent variable **y**. The aim of regression analysis is to estimate the parameters on the basis of available/observed empirical data. Traditional studies on regression assume the observations to have crisp values. In the crisp linear regression model, the parameters (regression coefficients are crisp) appear in a linear form; that is,

(1) $$\begin{array}{c} y=\beta_{0}+\beta_{1}x_{1}+\beta_{2}x_{2}+\cdots +\beta_{k}x_{k}+\text{random}\;\text{error}. \end{array}$$

Once the coefficients *β*_0_, *β*_1_, *β*_2_,…, *β*_*k*_ are determined from the observed samples, the responses are estimated from any given sets of **x**_1_, **x**_2_,…, **x**_*k*_ values.

Fuzzy set theory, developed by Zadeh [1], has capability to describe the uncertain situations, containing ambiguity and vagueness. It may be recalled that a fuzzy set *A* defined on a universe of discourse *X* is characterized by a membership function *μ*_*A*_(*x*) which takes values in the interval [0,1] (i.e., *μ*_*A*_ : *X* → [0,1]). The value *μ*_*A*_(*x*) represents the grade of membership of *x* ∈ *X* in *A*. This grade corresponds to the degree to which that element or individual is similar or compatible with the concept represented by the fuzzy set. Thus, the elements may belong in the fuzzy set to a greater or lesser degree as indicated by a larger or smaller membership grade.

Tanaka et al. [2, 3] initiated the research in the area of linear regression analysis in a fuzzy environment, where a fuzzy linear system is used as a regression model. They consider a regression model in which the relations of the variables are subject to fuzziness, that is, the model with crisp input and fuzzy parameters. In general, fuzzy regression can be classified into two categories:

- when the relations of the variables are subject to fuzziness,
- when the variables themselves are fuzzy.

There exist several conceptual and methodological approaches to fuzzy regression with respect to the characterization mentioned above. Tanaka and Watada [4], Tanaka et al. [5], and Tanaka and Ishibuchi [6] considered more general models in fuzzy regression. In the approaches of Tanaka et al., they considered the L-R fuzzy data and minimized the index of fuzziness of the fuzzy linear regression model. As described by Tanaka and Watada [4], “*A fuzzy number is a fuzzy subset of the real line whose highest membership values are clustered around a given real number called the mean value; the membership function is monotonic on both sides of this mean value*.” Hence, fuzzy number can be decomposed into position and fuzziness, where the position is represented by the element with the highest membership value and the fuzziness of a fuzzy number is represented by the membership function. The comparison among various fuzzy regression models and the difference between the approaches of fuzzy regression analysis and conventional regression analysis have been presented by Redden and Woodall [7]. Chang and Lee [8] and Redden and Woodall [7] pointed out some weaknesses of the approaches proposed by Tanaka et al. A fuzzy linear regression model based on Tanaka's approach by considering the fuzzy linear programming problem has also been introduced by Peters [9].

In fuzzy set theory, the entropy is a measure of degree of fuzziness which expresses the amount of average ambiguity/difficulty in making a decision whether an element belongs to a set or not. The following are the four properties introduced in de Luca and Termini [10], which are widely accepted as a criterion for defining any new fuzzy entropy measure *H*(·) of the fuzzy set *A*:

(i) P1 (*sharpness*): *H*(*A*) is minimum if and only if *A* is a crisp set; that is, *μ*_*A*_(*x*) = 0 or 1 for all *x*;
(ii) P2 (*maximality*): *H*(*A*) is maximum if and only if *μ*_*A*_(*x*) = 0.5 for all *x*;
(iii) P3 (*resolution*): *H*(*A*) ≥ *H*(*A* ^*^), where *A* ^*^ is sharpened version of *A*;
(iv) P4 (*symmetry*): $H(A)=H(\bar{A})$, where $\bar{A}$ is the complement of *A*; that is, $\mu_{\bar{A}}(x)=1-\mu_{A}(x)$.

Dubosis and Prade [11, 12] interpreted the measure of fuzziness *H*(*A*) as quantity of information which is being lost in going from a crisp number to a fuzzy number. It may be noted that the entropy of an element with a given membership function $\mu_{\tilde{A}}(x)$ is increasing if *μ*_*A*_(*x*) is in [0, 0.5] and decreasing if *μ*_*A*_(*x*) is in [0.5, 1]. We accept the definition of fuzzy number given by Tanaka and Watada [4], where the mean value is also called apex.

Let *X* = (*x*_1_, *x*_2_,…, *x*_*n*_) be a discrete random variable with probability distribution *P* = (*p*_1_, *p*_2_,…, *p*_*n*_) in an experiment; then according to Shannon [13], the information contained in this experiment is given by

(2) $$\begin{array}{c} H\left(P\right)=-\overset{n}{\underset{i=1}{\sum }}p_{i}\log p_{i}. \end{array}$$

Based on this famous Shannon's entropy, de Luca and Termini [10] indicated the following measure of fuzzy entropy:

(3) H(A)=−K∫x∈X[μA(x)log⁡μA(x)     +(1−μA(x))log⁡(1−μA(x))]dx.

Kumar et al. [14] studied fuzzy linear regression (FLR) model with some restrictions in the form of prior information and obtained the estimators of regression coefficients with the help of fuzzy entropy for the restricted FLR model. Here, we propose an intuitionistic fuzzy regression model and its general form in triangular intuitionistic fuzzy setup is given by

(4) $$\begin{array}{c} \tilde{y}={\tilde{\beta }}_{0}+{\tilde{\beta }}_{1}{\tilde{x}}_{1}+\cdots +{\tilde{\beta }}_{k}{\tilde{x}}_{k}+\text{random}\;\text{error}, \end{array}$$

where the value of the output variable $\tilde{y}$ defined by (4) is a triangular intuitionistic fuzzy number; ${\tilde{\beta }}_{0},{\tilde{\beta }}_{1},\ldots ,{\tilde{\beta }}_{k}$ is a vector of intuitionistic fuzzy parameters where ${\tilde{\beta }}_{j}=(m_{j};\alpha_{j},\beta_{j};\alpha_{j}^{\prime },\beta_{j}^{\prime })$ is a triangular intuitionistic fuzzy number for *j* = 0,1,…, *k* and ${\tilde{x}}_{1},{\tilde{x}}_{2},\ldots ,{\tilde{x}}_{k}$ are triangular intuitionistic fuzzy (explanatory) variables.

### 1.1. Intuitionistic Fuzzy Sets: Basic Definitions and Notations

It may be recalled that a fuzzy set *A* in *X*, given by Zadeh [1], is as follows:

(5) $$\begin{array}{c} A=\left\{\left(x,\mu_{A}\left(x\right)\right):x\in X\right\}, \end{array}$$

where *μ*_*A*_ : *X* → [0,1] is the membership function of the fuzzy set *A* and *μ*_*A*_(*x*) is the grade of belongingness of *x* into *A*. Thus in fuzzy set theory the grade of nonbelongingness of an element *x* into *A* is equal to 1 − *μ*_*A*_(*x*). However, while expressing the degree of membership of an element in a fuzzy set, the corresponding degree of nonmembership is not always equal to one minus the degree of belongingness. The fact is that, in real life, the linguistic negation does not always identify with logical negation. Therefore, Atanassov [15–18] suggested a generalization of classical fuzzy set, called intuitionistic fuzzy set (IFS).

Atanassov's IFS $\tilde{A}$ under the universal set *X* is defined as

(6) $$\begin{array}{c} \tilde{A}=\left\{\langle x,\mu_{\tilde{A}}\left(x\right),\nu_{\tilde{A}}\left(x\right)\rangle :x\in X\right\}, \end{array}$$

where $\mu_{A},\nu_{\tilde{A}}:X\to [0,1]$ are the membership and nonmembership functions such that $0\leq \mu_{\tilde{A}}+\mu_{\tilde{A}}\leq 1$ for all *x* ∈ *X*. The numbers $\mu_{\tilde{A}}(x)$ and $\nu_{\tilde{A}}(x)$ denote the degree of membership and nonmembership of an element *x* ∈ *X* to the set $\tilde{A}\subset X$, respectively. For each element *x* ∈ *X*, the amount $\pi_{\tilde{A}}(x)=1-\mu_{\tilde{A}}(x)-\nu_{\tilde{A}}(x)$ is called the degree of indeterminacy (hesitation part). It is the degree of uncertainty whether *x* belongs to $\tilde{A}$ or not.

### 1.2. Intuitionistic Fuzzy Numbers (IFNs)

In literature, Burillo and Bustince [19], Lee [20], Liu and Shi [21], and Grzegorzewski [22] proposed various research works on intuitionistic fuzzy numbers. In this section, the notion of IFNs has been studied and presented by the taking care of these research works.

**Definition 1 .**

An intuitionistic fuzzy subset $\tilde{A}=\left\{\langle x,\mu_{\tilde{A}}(x),\nu_{\tilde{A}}(x)\rangle :x\in X\right\}$ of the real line *ℝ* is called an intuitionistic fuzzy number if the following axioms hold:

(i) $\tilde{A}$ is normal; that is, there exist *m* ∈ *ℛ* (sometimes called the mean value of $\tilde{A}$) such that $\mu_{\tilde{A}}(m)=1$ and $\nu_{\tilde{A}}(m)=0$;
(ii) the membership function $\mu_{\tilde{A}}$ is fuzzy-convex; that is,
  (7) μA~(λ·x1+(1−λ)·x2)≥min⁡{μA~(x1),μA~(x2)}∀x1,x2∈X,  λ∈[0,1];
(iii) the nonmembership function $\nu_{\tilde{A}}$ is fuzzy-concave; that is
  (8) νA~(λ·x1+(1−λ)·x2)≤max⁡{νA~(x1),νA~(x2)},∀x1,x2∈X,  λ∈[0,1];
(iv) the membership and the nonmembership functions of $\tilde{A}$ satisfying the conditions 0 ≤ *f*_1_(*x*) + *g*_1_(*x*) ≤ 1 and 0 ≤ *f*_2_(*x*) + *g*_2_(*x*) ≤ 1 have the following form:
  (9) $$\begin{array}{c} \mu_{\tilde{A}}\left(x\right)=\{\begin{array}{cc} f_{1}\left(x\right), & \text{for}\;m-\alpha \leq x\leq m,\\ 1, & \text{for}\;x=m,\\ f_{2}\left(x\right), & \text{for}\;m\leq x\leq m+\beta ,\\ 0, & \text{otherwise}, \end{array} \end{array}$$
 where the functions *f*_1_(*x*) and *f*_2_(*x*) are strictly increasing and decreasing functions in [*m* − *α*, *m*] and [*m*, *m* + *β*], respectively, and
  (10) $$\begin{array}{c} \nu_{\tilde{A}}\left(x\right)=\{\begin{array}{cc} g_{1}\left(x\right), & \text{for}\;m-\alpha^{\prime }\leq x\leq m,\\ 0, & \text{for}\;x=m,\\ g_{2}\left(x\right), & \text{for}\;m\leq x\leq m+\beta^{\prime },\\ 1, & \text{otherwise}, \end{array} \end{array}$$
 where the functions *g*_1_(*x*) and *g*_2_(*x*) are strictly decreasing and increasing functions in [*m* − *α*′, *m*] and [*m*, *m* + *β*′], respectively. Here *α* and *β* are called the left and right spreads of the membership function $\mu_{\tilde{A}}$, respectively. *α*′ and *β*′ are called the left and right spreads of the nonmembership function $\nu_{\tilde{A}}(x)$. Symbolically, an intuitionistic fuzzy number is represented as ${\tilde{A}}_{\text{IFN}}=\left(m;\alpha ,\beta ;\alpha^{\prime },\beta^{\prime }\right)$.

**Definition 2 .**

An IFN *A*_IFN_ = (*m*; *α*, *β*; *α*′, *β*′) may be defined as a triangular intuitionistic fuzzy number (TIFN) if and only if its membership and nonmembership functions take the following form:

(11) μA~(x)={1−m−xα,for  m−α≤x≤m,1,for  x=m,1−x−mβ,for  m≤x≤m+β,0,otherwise,

(12) νA~(x)={m−xα′,for  m−α′≤x≤m,0,for  x=m,x−mβ′,for  m≤x≤m+β′,1,otherwise.

It may be noted that a TIFN $\tilde{A}=(m;\alpha ,\beta ;\alpha^{\prime },\beta^{\prime })$ degenerate to a triangular fuzzy number *A* = (*m*; *α*, *β*) if *α* = *α*′, *β* = *β*′, and $\nu_{\tilde{A}}(x)=1-\mu_{\tilde{A}}(x)$, ∀*x* ∈ *ℛ*. Further, an TIFN $\tilde{A}=\left\{\langle x,\mu_{\tilde{A}}(x),\nu_{\tilde{A}}(x)\rangle :x\in ℛ\right\}$; that is, $\tilde{A}=\left(m;\alpha ,\beta ;\alpha^{\prime },\beta^{\prime }\right)$ is a conjunction of two fuzzy numbers *A* ^+^ = (*m*; *α*, *β*) with the membership function $\mu_{A^{+}}(x)=\mu_{\tilde{A}}(x)$ and *A* ^−^ = (*m*; *α*′, *β*′) with the membership function $\mu_{\tilde{A}}(x)=1-\nu_{\tilde{A}}(x)$.

The entropy calculated using (3) from the membership function of TIFN given by (11) can be expressed as follows: size

(13) H(A¯)=−K[∫x∈[m−α,m][μA¯(x)log⁡μA¯(x)+(1−μA¯(x))          ×log⁡⁡(1−μA¯(x))]dx  +∫x∈[m,m+β][μA¯(x)log⁡μA¯(x)+(1−μA¯(x))          ×log⁡⁡(1−μA¯(x))]dx∫x∈[m−α,m][μA¯(x)log⁡μA¯(x)+(1−μA¯(x))]=HL(A~)+HR(A~),

where $H_{L}(\tilde{A})=K\alpha /2$ and $H_{R}(\tilde{A})=K\beta /2$. It follows that $H(\tilde{A})=K(\alpha +\beta )/2$, which does not depend on *m*. It may be observed that, in the case of symmetrical TIFN, the left and the right entropies are identical. On the other hand, in case of nonsymmetric TIFN, the left entropy is a function of *α* and the right entropy is a function of *β*. Similarly, the left entropy and the right entropy from the nonmembership function (which we called left to left and right to right entropies) of the TIFN are the functions of *α*′ and *β*′, respectively. Hence, a triangular intuitionistic fuzzy number can be characterized by five attributes: the position parameter *m*, the left entropy *α*, the right entropy *β*, left to left entropy *α*′, and right to right entropy *β*′. There is a one-to-one correspondence between a triangular intuitionistic fuzzy number and its entropies. In other words, given a triangular intuitionistic fuzzy number, one can determine the unique position and entropies. Conversely, given a position and entropies, one can construct a unique triangular intuitionistic fuzzy number.

Sometimes experimenter's past experiences may be available as prior information about unknown regression coefficients to estimate more efficient estimators. Here, we assume that such prior information is provided in the form of exact linear restrictions on regression coefficients. In the present work, we first find the unrestricted estimators of regression coefficients with the help of fuzzy entropy. Next, we introduce the restricted intuitionistic fuzzy linear regression model with fuzzy entropy. Further, the restricted estimators of the regression coefficients are obtained by incorporating the prior information in the form of linear restrictions.

## 2. Restricted IFWLR Model with Fuzzy Entropy

Without loss of generality, suppose that all observations (${\tilde{y}}_{i},{\tilde{x}}_{i1},{\tilde{x}}_{i2},\ldots ,{\tilde{x}}_{ik}$), *i* = 1,…, *n*, in the regression analysis are triangular intuitionistic fuzzy numbers. The notion of regression using fuzzy entropy is to construct five conventional regression equations (one for apex, one for left entropy of the membership function, one for right entropy of the membership function, one for left entropy of the nonmembership function, and one for right entropy of the nonmembership function) for the response variable $\tilde{y}$ using the corresponding attributes of the *k* fuzzy explanatory variables ${\tilde{x}}_{j}$. In order to be specific, we denote **y** ^**a**^, **x**_1_^**a**^, **x**_2_^**a**^,…, **x**_*k*_^**a**^ by the apexes of $\tilde{y},{\tilde{x}}_{1},{\tilde{x}}_{2},\ldots ,{\tilde{x}}_{k}$, respectively, **e**_**y**_^**l**^, **e**_**x**_1__^**l**^, **e**_**x**_2__^**l**^,…, **e**_**x**_*k*__^**l**^ by the left entropy of $\tilde{y},{\tilde{x}}_{1},{\tilde{x}}_{2},\ldots ,{\tilde{x}}_{k}$, respectively, **e**_**y**_^**r**^, **e**_**x**_1__^**r**^, **e**_**x**_2__^**r**^,…, **e**_**x**_*k*__^**r**^ by the right entropy of $\tilde{y},{\tilde{x}}_{1},{\tilde{x}}_{2},\ldots ,{\tilde{x}}_{k}$, respectively, **e**_**y**_^**l**′^, **e**_**x**_1__^**l**′^, **e**_**x**_2__^**l**′^,…, **e**_**x**_*k*__^**l**′^ by the left to left entropy of $\tilde{y},{\tilde{x}}_{1},{\tilde{x}}_{2},\ldots ,{\tilde{x}}_{k}$, respectively, and **e**_**y**_^**r**′^, **e**_**x**_1__^**r**′^, **e**_**x**_2__^**r**′^,…, **e**_**x**_*k*__^**r**′^ by the right to right entropy of $\tilde{y},{\tilde{x}}_{1},{\tilde{x}}_{2},\ldots ,{\tilde{x}}_{k}$, respectively. Therefore, the five fundamental regression equations in a nonrecursive (nonadaptive) setup may be written as

(14) ya=A0a+∑i=1k(Aiaxia+Biaexil+Ciaexir+Diaexil′+Eiaexir′)+ɛya;eyl=A0l+∑i=1k(Ailxia+Bilexil+Cilexir+Dilexil′+Eilexir′)+ɛeyl;eyr=A0r+∑i=1k(Airxia+Birexil+Cirexir+Direxil′+Eirexir′)+ɛeyr;eyl′=A0l′+∑i=1k(Ail′xia+Bil′exil+Cil′exir+Dil′exil′+Eil′exir′)+ɛeyl′;eyr′=A0r′+∑i=1k(Air′xia+Bir′exil+Cir′exir+Dir′exil′+Eir′exir′)+ɛeyr′,

where **ɛ**_**y**^**a**^_, **ɛ**_**e**_**y**_^**l**^_, **ɛ**_**e**_**y**_^**r**^_, **ɛ**_**e**_**y**_^**l**′^_, and **ɛ**_**e**_**y**_^**r**′^_ are the error vectors of dimension *n* × 1. The compact form of the above mentioned nonrecursive or nonadaptive equations is given by

(15) $$\begin{array}{c} y^{a}=X\beta +{ɛ}_{y^{a}},\\ e_{y}^{l}=X\alpha +{ɛ}_{e_{y}^{l}},\\ e_{y}^{r}=X\gamma +{ɛ}_{e_{y}^{r}},\\ e_{y}^{l^{\prime }}=X\alpha^{\prime }+{ɛ}_{e_{y}^{l^{\prime }}},\\ e_{y}^{r^{\prime }}=X\gamma^{\prime }+{ɛ}_{e_{y}^{r^{\prime }}}, \end{array}$$

where

(16) X=(1  ⋮  x1a,x2a,…,xka  ⋮  ex1l,ex2l,…,exkl  ⋮  ex1r, ex2r,…,exkr  ⋮  ex1l′,ex2l′,…,exkl′  ⋮  ex1r′,ex2r′,…,exkr′)n×(5k+1),β=(A0a  ⋮  A1a,A2a,…,Aka  ⋮  B1a,B2a,…,Bka  ⋮  C1a,C2a,…, Cka  ⋮  D1a,D2a,…,Dka  ⋮  E1a,E2a,…,Eka)(5k+1)×1T,α=(A0l  ⋮  A1l,A2l,…,Akl  ⋮  B1l,B2l,…,Bkl  ⋮  C1l,C2l,…, Ckl  ⋮  D1l,D2l,…,Dkl  ⋮  E1l,E2l,…,Ekl)(5k+1)×1T,γ=(A0r  ⋮  A1r,A2r,…,Akr  ⋮  B1r,B2r,…,Bkr  ⋮  C1r,C2r,…, Ckr  ⋮  D1r,D2r,…,Dkr  ⋮  E1r,E2r,…,Ekr)(5k+1)×1T,α′=(A0l′  ⋮  A1l′,A2l′,…,Akl′  ⋮  B1l′,B2l′,…,Bkl′  ⋮  C1l′,C2l′,…, Ckl′  ⋮  D1l′,D2l′,…,Dkl′  ⋮  E1l′,E2l′,…,Ekl′)(5k+1)×1T,γ′=(A0r′  ⋮  A1r′,A2r′,…,Akr′  ⋮  B1r′,B2r′,…,Bkr′  ⋮  C1r′,C2r′,…, Ckr′  ⋮  D1r′,D2r′,…,Dkr′  ⋮  E1r′,E2r′,…,Ekr′)(5k+1)×1T.

In many real life situations, where the measurements are carried out (for example car speed astronomical distance), it is natural to think that the spread (vagueness) in the measure of a phenomenon is proportional to its intensity. D'Urso and Gastaldi [23] have done several simulations and observed that even if we consider an adaptive or recursive regression model along with nonadaptive or nonrecursive regression model, they yield identical solutions when there is only one independent variable. But if there are more than one independent variable, then the estimated values of the left entropies and right entropies obtained through the recursive fuzzy regression model will have less variance as compared to the nonrecursive fuzzy regression model. With this consideration, we rewrite the proposed intuitionistic fuzzy linear regression model (15) in a recursive/adaptive setup where dynamic of the entropies is dependent on the magnitude of the estimated apexes as follows:

(17) $$\begin{array}{c} y^{a}=y^{a^{*}}+{ɛ}_{y^{a}};\;\text{where}\;\;y^{a^{*}}=X\beta ,\\ e_{y}^{l}=e_{y}^{l^{*}}+{ɛ}_{e_{y}^{l}}^{*};\;\text{where}\;\;e_{y}^{l^{*}}=X\beta b+1d,\\ e_{y}^{r}=e_{y}^{r^{*}}+{ɛ}_{e_{y}^{r}}^{*};\;\text{where}\;\;e_{y}^{r^{*}}=X\beta f+1g,\\ e_{y}^{l^{\prime }}=e_{y}^{{l^{\prime }}^{*}}+{ɛ}_{e_{y}^{l^{\prime }}}^{*};\;\text{where}\;\;e_{y}^{{l^{\prime }}^{*}}=X\beta p+1q,\\ e_{y}^{r^{\prime }}=e_{y}^{{r^{\prime }}^{*}}+{ɛ}_{e_{y}^{r^{\prime }}}^{*};\;\text{where}\;\;e_{y}^{{r^{\prime }}^{*}}=X\beta u+1v, \end{array}$$

where **X** is the *n* × (5*k* + 1)-matrix containing the values of the input variables (data matrix), **β** is a column 5*k* + 1-vector containing the regression coefficients for the apexes of the first model (referred to as core regression model), **y** ^**a**^ and **y** ^a^^*^ are the vector of the observed apexes and the vector of the interpolated apexes, respectively, both having dimension *n* × 1, **e**_**y**_^**l**^ and **e**_**y**_^l^^*^ are the vector of the observed left entropies and the vector of the interpolated left entropies, respectively, both having dimension *n* × 1, **e**_**y**_^**r**^ and **e**_**y**_^r^^*^ are the vector of the observed right entropies and the vector of the interpolated right entropies, respectively, both having dimension *n* × 1, **e**_**y**_^**l**′^ and **e**_**y**_^**l**′^^*^ are the vector of the observed left to left entropies and the vector of the interpolated left to left entropies, respectively, both having dimension *n* × 1, **e**_**y**_^**r**′^ and **e**_**y**_^**r**′^^*^ are the vector of the observed right to right entropies and the vector of the interpolated right to right entropies, respectively, both having dimension *n* × 1, and** 1** is a (*n* × 1)-vector of all 1′s, *b* and *d* are regression parameters for the second regression equation model (referred to as left entropy regression model), *f* and *g* are regression parameters for the third regression model (referred to as right entropy regression model), *p* and *q* are regression parameters for the fourth regression equation model (referred to as left to left entropy regression model), and *u* and *v* are regression parameters for the fifth regression equation model (referred to as right to right entropy regression model). The error term in the regression equation of apexes will remain the same while the error terms in the regression equations of entropies may be different. The error vectors **ɛ**_**e**_**y**_^l^_^*^ and **ɛ**_**e**_**y**_^r^_^*^ in the left and right entropies are of the dimension (*n* × 1) and the error vectors **ɛ**_**e**_**y**_^l^_^*^ and **ɛ**_**e**_**y**_^r^_^*^ in the left to left and right to right entropies are of the dimension (*n* × 1).

If some prior information about unknown regression coefficients is available on the basis of past experiences, then it may be used to estimate more efficient estimators. We assume that such prior information is in the form of exact linear restrictions on regression coefficients. In the present model, we associate such restrictions in the equations for the estimation of regression coefficients in the intuitionistic fuzzy linear regression model with fuzzy entropy. Therefore, we make the model capable of taking into account possible linear relations between the size of the entropies and the magnitude of the estimated apexes. Moreover, we assume that the regression coefficients **β** are subjected to the *j* (*j* < 5*k* + 1) exact linear restrictions, which are given by

(18) $$\begin{array}{c} h=H\beta , \end{array}$$

where **h** and **H** are known and the matrix **H** is of full row rank.

## 3. Estimation of Regression Coefficients

In many applications, it is possible that the values of the variables are on completely different scales of measurement. Also, the possible larger variations in the values will have larger intersample differences, so they will dominate in the calculation of Euclidean distances. Therefore, some form of standardization is necessary to balance out the individual contributions. Consider the Euclidean distance between two triangular intuitionistic fuzzy numbers *y*_*i*_ = (*y*_*i*_^*a*^; *e*_*y*_*i*__^*l*^, *e*_*y*_*i*__^*r*^; *e*_*y*_*i*__^*l*′^, *e*_*y*_*i*__^*r*′^) and *y*_i_^*^ = (*y*_*i*_^a^*^^; *e*_*y*_*i*__^l^*^^, *e*_*y*_*i*__^r^*^^; *e*_*y*_*i*__^*l*′^*^^, *e*_*y*_*i*__^*r*′^*^^) along with weights *w*_1_, *w*_2_, *w*_3_, *w*_4_, and *w*_5_ as follows:

(19) δi≡δ(yi,yi∗)=((eyil′−eyil′∗)2w1(yia−yia∗)2+w2(eyil−eyil∗)2+w3(eyir−eyir∗)2  +w4(eyil′−eyil′∗)2+w5(eyir′−eyir′∗)2)1/2.

It may be observed that we compute the usual squared differences between the values of variables on their original scales, as in the usual Euclidean distance, but then multiply these squared differences by their corresponding weights.

Next, similar to common linear regression (based on crisp data), the regression parameters are estimated by minimizing the following sum of square errors (we use a compact matrix notation):

(20) φ(β,b,d,f,g,p,q,u,v) =∑i=1nw1(yia−yia∗)2+∑i=1nw2(eyil−eyil∗)2  +∑i=1nw3(eyir−eyir∗)2+∑i=1nw4(eyil′−eyil′∗)2  +∑i=1nw5(eyir′−eyir′∗)2 =w1(ya−ya∗)T(ya−ya∗)+w2(eyl−eyl∗)T(eyl−eyl∗)  +w3(eyr−eyr∗)T(eyr−eyr∗)  +w4(eyl′−eyl′∗)T(eyl′−eyl′∗)  +w5(eyr′−eyr′∗)T(eyr′−eyr′∗) =w1((ya)Tya−2(ya)Tya∗+(ya∗)Tya∗)  +w2((eyl)Teyl−2(eyl)Teyl∗+(eyl∗)Teyl∗)  +w3((eyr)Teyr−2(eyr)Teyr∗+(eyr∗)Teyr∗)  +w4((eyl′)Teyl′−2(eyl′)Teyl′∗+(eyl′∗)Teyl′∗)  +w5((eyr′)Teyr′−2(eyr′)Teyr′∗+(eyr′∗)Teyr′∗) =w1((ya)Tya−2(ya)TXβ+βTXTXβ)  +w2((eyl)Teyl−2(eyl)T(Xβb+1d))  +w2((Xβb+1d)T(Xβb+1d))  +w3((eyr)Teyr−2(eyr)T(Xβf+1g))  +w3((Xβf+1g)T(Xβf+1g))  +w4((eyl′)Teyl′−2(eyl′)T(Xβp+1q))  +w4((Xβp+1q)T(Xβp+1q))  +w5((eyr′)Teyr′−2(eyr′)T(Xβu+1v))  +w5((Xβu+1v)T(Xβu+1v)) =w1((ya)Tya−2(ya)TXβ)  +βTXTXβ(w1+w2b2+w3f2+w4p2+w5u2)  +w2((eyl)Teyl−2(eyl)TXβb−2(eyl)T1d)  +w3((eyr)Teyr−2(eyr)TXβf−2(eyr)T1g)  +w4((eyl′)Teyl′−2(eyl′)TXβp−2(eyl′)T1q)  +w5((eyr′)Teyr′−2(eyr′)TXβu−2(eyr′)T1v)  +2βTXT1(w2bd+w3fg+w4pq+w5uv)  +n(w2d2+w3g2+w4q2+w5v2).

Differentiating *φ*(**β**, *b*, *d*, *f*, *g*, *p*, *q*, *u*, *v*), that is, (20), partially with respect to **β** and equating it to zero, we get

(21) ∂φ(β,b,d,f,g,p,q,u,v)∂β=0⟹−w1XTya+XTXβ(w1+w2b2+w3f2+w4p2+w5u2)⟹−w2XTeylb−w3XTeyrf−w4XTeyl′p−w5XTeyr′u⟹+XT1(w2bd+w3fg+w4pq+w5uv)=0⟹β=((XTX)−1XT[w1ya+w2eylb+w3eyrf⟹         +w4eyl′p+w5eyr′u⟹         −1(w2bd+w3fg+w4pq+w5uv)eyl](XTX)−1)⟹  ×(w1+w2b2+w3f2+w4p2+w5u2)−1.

Similarly, differentiating (20) partially with respect to *b*, *d*, *f*, *g*, *p*, *q*, *u*, and *v*, we get

(22) $$\begin{array}{c} b={\left(\beta^{T}X^{T}X\beta \right)}^{-1}\left[{\left(e_{y}^{l}\right)}^{T}X\beta -\beta^{T}X^{T}1d\right]; \end{array}$$

(23) $$\begin{array}{c} d=\frac{1}{n}\left[{\left(e_{y}^{l}\right)}^{T}1-\beta^{T}X^{T}1b\right]; \end{array}$$

(24) $$\begin{array}{c} f={\left(\beta^{T}X^{T}X\beta \right)}^{-1}\left[{\left(e_{y}^{r}\right)}^{T}X\beta -\beta^{T}X^{T}1g\right]; \end{array}$$

(25) $$\begin{array}{c} g=\frac{1}{n}\left[{\left(e_{y}^{r^{\prime }}\right)}^{T}1-\beta^{T}X^{T}1f\right]; \end{array}$$

(26) $$\begin{array}{c} p={\left(\beta^{T}X^{T}X\beta \right)}^{-1}\left[{\left(e_{y}^{l^{\prime }}\right)}^{T}X\beta -\beta^{T}X^{T}1q\right]; \end{array}$$

(27) $$\begin{array}{c} q=\frac{1}{n}\left[{\left(e_{y}^{l^{\prime }}\right)}^{T}1-\beta^{T}X^{T}1p\right]; \end{array}$$

(28) $$\begin{array}{c} u={\left(\beta^{T}X^{T}X\beta \right)}^{-1}\left[{\left(e_{y}^{r^{\prime }}\right)}^{T}X\beta -\beta^{T}X^{T}1v\right]; \end{array}$$

(29) $$\begin{array}{c} v=\frac{1}{n}\left[{\left(e_{y}^{r^{\prime }}\right)}^{T}1-\beta^{T}X^{T}1u\right]; \end{array}$$

respectively.

Equations (21)–(29) are recursive solutions for the problem of least square estimation with intuitionistic fuzzy data. Therefore, we rewrite the system of equations explicitly in a recursive way as follows:

(30) βi+1=((XTX)−1XT[w1ya+w2eylbi+w3eyrfi+w4eyl′pi       +w5eyr′ui−1(w2bidi+w3figi              +w4piqi+w5uivi)w1ya+w2eylbi+w3eyrfi+w4eyl′pi](XTX)−1)×(w1+w2bi2+w3fi2+w4pi2+w5ui2)−1;bi+1=(βi+1TXTXβi+1)−1[(eyl)TXβi+1−βi+1TXT1di];di+1=1n[(eyl)T1−βi+1TXT1bi];fi+1=(βi+1TXTXβi+1)−1[(eyr)TXβi+1−βi+1TXT1gi];gi+1=1n[(eyr′)T1−βi+1TXT1fi];pi+1=(βi+1TXTXβi+1)−1[(eyl′)TXβi+1−βi+1TXT1qi];qi+1=1n[(eyl′)T1−βi+1TXT1pi];ui+1=(βi+1TXTXβi+1)−1[(eyr′)TXβi+1−βi+1TXT1vi];vi+1=1n[(eyr′)T1−βi+1TXT1ui].

In order to initiate the recursive process of obtaining the estimators, we take some initial values for *b*, *d*, *f*, *g*, *p*, *q*, *u*, *v*, and **β**. After several numbers of iterations, the values of estimators get corrected to a predefined error of tolerance. We denote these values by $\hat{b}$, $\hat{d}$, $\hat{f}$, $\hat{g}$, $\hat{p}$, $\hat{q}$, $\hat{u}$, $\hat{v}$, and $\hat{\beta }$ in order to differentiate them from the eventually obtained restricted estimator $\tilde{\beta }$ in the next commutation.

In a more general setup, if, in the linear regression model (17), we consider *k*_1_ crisp and *k*_2_ intuitionistic fuzzy input variables, then the dimensions of **X** and **β** will be *n* × (*k*_1_ + 5*k*_2_ + 1) and (*k*_1_ + 5*k*_2_ + 1) × 1, respectively. It may further be noted that the core of the solution's structure will remain the same and we will have similar kind of estimators.

*Remark.* If a TIFN $\tilde{A}=(m;\alpha ,\beta ;\alpha^{\prime },\beta^{\prime })$ degenerate to a triangular fuzzy number *A* = (*m*; *α*, *β*), then our nonsymmetric intuitionistic fuzzy weighted linear regression model reduces to nonsymmetric fuzzy linear regression model defined by Kumar et al. [24].

Next, we assume that the regression coefficients are subjected to the linear restrictions which are given by (18). It may be noted that the unrestricted estimator obtained above in (21) does not satisfy the given restrictions (18). We aim to obtain the restricted estimator which satisfies the given restrictions under the regression model (17). For this, we propose to minimize the following score function:

(31) S(λ,β,b,d,f,g,p,q,u,v) =φ(β,b,d,f,g,p,q,u,v)−2λ(Hβ−h) =w1((ya)Tya−2(ya)TXβ)  +βTXTXβ(w1+w2b2+w3f2+w4p2+w5u2)  +w2((eyl)Teyl−2(eyl)TXβb−2(eyl)T1d)  +w3((eyr)Teyr−2(eyr)TXβf−2(eyr)T1g)  +w4((eyl′)Teyl′−2(eyl′)TXβp−2(eyl′)T1q)  +w5((eyr′)Teyr′−2(eyr′)TXβu−2(eyr′)T1v)  +2βTXT1(w2bd+w3fg+w4pq+w5uv)  +n(w2d2+w3g2+w4q2+w5v2)  −2λ(Hβ−h),

where 2*λ* is the vector of Lagrange's Multiplier. 

Differentiating *S*(*λ*, **β**, *b*, *d*, *f*, *g*, *p*, *q*, *u*, *v*) partially with respect to **β** and equating it to zero, we get

(32) ⟹−w1XTya+XTXβ(w1+w2b2+w3f2+w4p2+w5u2)   −w2XTeylb−w3XTeyrf−w4XTeyl′p−w5XTeyr′u   +XT1(w2bd+w3fg+w4pq+w5uv)−H′λ=0.

Here, we again relabel the computed restricted estimator by $\tilde{\beta }$. Therefore, in view of (21) and (32), we get size

(33) ⟹β~=((XTX)−1XT[w1ya+w2eylb+w3eyrf+w4eyl′p          +w5eyr′u          −1(w2bd+w3fg+w4pq+w5uv)eyl′](XTX)−1)   ×(w1+w2b2+w3f2+w4p2+w5u2)−1   +(XTX)−1HTλ(w1+w2b2+w3f2+w4p2+w5u2)⟹β~=β^+1(w1+w2b2+w3f2+w4p2+w5u2)    ×(XTX)−1HTλ.

Similarly, differentiating *S*(*λ*, **β**, *b*, *d*, *f*, *g*, *p*, *q*, *u*, *v*) partially with respect to *λ* and equating it to zero, we get

(34) ⟹Hβ~=h⟹Hβ^+1(w1+w2b2+w3f2+w4p2+w5u2)  ×H(XTX)−1HTλ=h⟹λ^=(w1+w2b2+w3f2+w4p2+w5u2)    ×[H(XTX)−1HT]−1(h−Hβ^).

From (33) and (34), we have

(35) $$\begin{array}{c} ⟹\tilde{\beta }=\hat{\beta }+{\left(X^{T}X\right)}^{-1}H^{T}{\left[H{\left(X^{T}X\right)}^{-1}H^{T}\right]}^{-1}\left(h-H\hat{\beta }\right). \end{array}$$

Also, differentiating (31) partially with respect to *b*, *d*, *f*, *g*, *p*, *q*, *u*, and *v* and equating all to zero, we get

(36) $$\begin{array}{c} \tilde{b}=\hat{b},\;\;\tilde{d}=\hat{d},\;\;\tilde{f}=\hat{f},\;\;\tilde{g}=\hat{g},\\ \tilde{p}=\hat{p},\;\;\tilde{q}=\hat{q},\;\;\tilde{u}=\hat{u},\;\;\tilde{v}=\hat{v}, \end{array}$$

respectively. From (35) we see that

(37) ⟹Hβ~=Hβ^+[H(XTX)−1HT][H(XTX)−1HT]−1    ×(h−Hβ^)⟹Hβ~=Hβ^+(h−Hβ^)=h.

Therefore, the estimator $\tilde{\beta }$ satisfies the given restrictions (18).

## 4. Numerical Examples

We consider the following numerical examples to illustrate the proposed model.

**Example 1 .**

We apply our procedure to estimate the intuitionistic fuzzy output value for a data consisting of the crisp input and intuitionistic fuzzy output (where left entropy and right entropy are equal) and tabulate the data in Table 1.

We obtain $\hat{\beta }=(-4.4026,3.5733,7.3786,5.6858{)}^{\prime }$, $\hat{b}=0.2942$, $\hat{d}=14.7144$, $\hat{f}=0.2942$, $\hat{g}=14.7144$, $\hat{p}=0.2909$, $\hat{q}=17.4487$, $\hat{u}=0.2909$, and $\hat{v}=17.4487$ where the number of iterations required is 125.

**Example 2 .**

We apply our procedure to estimate intuitionistic fuzzy output value for a data consisting of crisp input and intuitionistic fuzzy output (where left and right entropy are not equal) and tabulate the data in Table 2.

We obtain $\hat{\beta }=(-4.7697,3.5933,7.2030,5.9152{)}^{\prime }$, $\hat{b}=0.2952$, $\hat{d}=14.5871$, $\hat{f}=0.2646$, $\hat{g}=20.3429$, $\hat{p}=0.3052$, $\hat{q}=15.7050$, $\hat{u}=0.2717$, and $\hat{v}=23.1201$ where the number of iterations required is 113.

**Example 3 .**

We apply our procedure to estimate intuitionistic fuzzy output value for a data consisting of crisp input, intuitionistic fuzzy input, and intuitionistic fuzzy output (where left and right entropy are not equal) and tabulate the data in Table 3.

We obtain $\hat{\beta }=(-3.2352,0.6811,0.5314,-0.9164,0.0846,-3.1631,2.953{)}^{\prime }$, $\hat{b}=0.4225$, $\hat{d}=0.5478$, $\hat{f}=0.4307$, $\hat{g}=0.1637$, $\hat{p}=0.3231$, $\hat{q}=3.8723$, $\hat{u}=0.4985$, and $\hat{v}=1.8659$ where the number of iterations required is 51.

**Example 4 .**

We apply our procedure to estimate intuitionistic fuzzy output value for a data consisting of intuitionistic fuzzy input and intuitionistic fuzzy output (where left and right entropy are not equal) and tabulate the data in Table 4.

We obtain $\hat{\beta }=(11.8141,-0.2161,1.6104,-1.8254,0.5687,-0.1879{)}^{\prime }$, $\hat{b}=0.3880$, $\hat{d}=0.3674$, $\hat{f}=0.3880$, $\hat{g}=0.3674$, $\hat{p}=0.3547$, $\hat{q}=2.2108$, $\hat{u}=0.3547$, and $\hat{v}=3.2108$ where the number of iterations required is 255.

## 5. Conclusions

An intuitionistic fuzzy weighted linear regression (IFWLR) model with and without some linear restrictions in the form of prior information has been studied. The estimators of regression coefficients have also been obtained with the help of fuzzy entropy for the restricted/unrestricted IFWLR model by assigning some weights in the distance function. It has been observed that the restricted estimator is better than unrestricted estimator in some sense. Thus, whenever some prior information is available in terms of exact linear restrictions on regression coefficients, it is advised to use restricted estimator $\tilde{\beta }$ in place of unrestricted estimator $\hat{\beta }$.

## Figures and Tables

**Table 1:** Crisp input-int. fuzzy output data.

Object *i*	Crisp input **X** = (**x**_1_, **x**_2_, **x**_3_)			Int. fuzzy output **y** = (**e**_**y**_^1′^, **e**_**y**_^1^, **y** ^**a**^, **e**_**y**_^**r**^, **e**_**y**_^**r**′^)					Estimated int. fuzzy output **y** ^*^ = (**e**_**y**_^1^∗′^^, **e**_**y**_^1^*^^, **y** ^a^*^^, **e**_**y**_^r^*^^, **e**_**y**_^**r**^∗′^^)				
	**x**_1_	**x**_2_	**x**_3_	**e**_**y**_^1′^	**e**_**y**_^1^	**y** ^**a**^	**e**_**y**_^**r**^	**e**_**y**_^**r**′^	**e**_**y**_^1^∗′^^	**e**_**y**_^1^*^^	**y** ^a^*^^	**e**_**y**_^r^*^^	**e**_**y**_^**r**^∗′^^
1	**3**	**5**	**9**	44	42	96	42	44	44.9018	42.4850	94.3828	42.4850	44.9018
2	**14**	**8**	**3**	48	47	120	47	48	52.8505	50.5256	121.7099	50.5256	52.8505
3	**7**	**1**	**4**	35	33	52	33	35	32.2052	29.6416	50.7324	29.6416	32.2052
4	**11**	**7**	**3**	50	45	106	45	50	47.5861	45.2004	103.6114	45.2004	47.5861
5	**7**	**12**	**15**	80	79	189	79	80	74.0058	71.9256	194.4413	71.9256	74.0058
6	**8**	**15**	**10**	68	65	194	65	68	73.2147	71.1253	191.7213	71.1253	73.2147
7	**3**	**9**	**6**	45	42	107	42	45	48.5252	46.1503	106.8398	46.1503	48.5252
8	**12**	**15**	**11**	80	78	216	78	80	79.0260	77.0038	211.7003	77.0038	79.0260
9	**10**	**5**	**8**	55	52	108	52	55	50.5235	48.1717	113.7100	48.1717	50.5235
10	**9**	**7**	**4**	45	44	103	44	45	47.1612	44.7706	102.1507	44.7706	47.1612

**Table 2:** Crisp input-int. fuzzy output data.

Object *i*	Crisp input **X** = (**x**_1_, **x**_2_, **x**_3_)			Int. fuzzy output **y** = (**e**_**y**_^1′^, **e**_**y**_^1^, **y** ^**a**^, **e**_**y**_^**r**^, **e**_**y**_^**r**′^)					Estimated int. fuzzy output **y** ^*^ = (**e**_**y**_^1^∗′^^, **e**_**y**_^1^*^^, **y** ^a^*^^, **e**_**y**_^r^*^^, **e**_**y**_^**r**^∗′^^)				
	**x**_1_	**x**_2_	**x**_3_	**e**_**y**_^1′^	**e**_**y**_^1^	**y** ^**a**^	**e**_**y**_^**r**^	**e**_**y**_^**r**′^	**e**_**y**_^1^∗′^^	**e**_**y**_^1^*^^	**y** ^a^*^^	**e**_**y**_^r^*^^	**e**_**y**_^**r**^∗′^^
1	**3**	**5**	**9**	45	42	96	47	48	44.7743	42.7104	95.2620	45.5472	49.0053
2	**14**	**8**	**3**	48	47	120	43	45	52.5995	50.2809	120.905	52.3320	55.9734
3	**7**	**1**	**4**	35	33	52	50	55	31.3430	29.7162	51.2469	33.9018	37.0452
4	**11**	**7**	**3**	46	45	106	45	47	47.1120	44.9720	102.922	47.5741	51.0870
5	**7**	**12**	**15**	82	79	189	80	85	75.3765	72.3166	195.547	72.0805	76.2555
6	**8**	**15**	**10**	70	65	194	60	67	74.0419	71.0254	191.173	70.9234	75.0671
7	**3**	**9**	**6**	45	42	107	40	46	48.1512	45.9774	106.328	48.4752	52.0124
8	**12**	**15**	**11**	80	78	216	88	90	80.2328	77.0149	211.461	76.2912	80.5799
9	**10**	**5**	**8**	55	52	108	50	55	50.6447	48.3897	114.499	50.6370	54.2327
10	**9**	**7**	**4**	45	44	103	42	44	46.7241	44.5967	101.651	47.2377	50.7415

**Table 3:** Crisp and int. fuzzy input-int. fuzzy output data.

Object *i*	Crisp and int. fuzzy input **X** = (**x**_1_, **e**_**x**_1__^1**'**^, **e**_**x**_1__^1^, **x**_1_^**a**^, **e**_**x**_1__^**r**^, **e**_**x**_1__^**r'**^)						Int. fuzzy output **y** = (**e**_**y**_^1′^, **e**_**y**_^1^, **y** ^**a**^, **e**_**y**_^**r**^, **e**_**y**_^**r**′^)					Estimated int. fuzzy output **y** ^*^ = (**e**_**y**_^1^∗′^^, **e**_**y**_^1^*^^, **y** ^a^*^^, **e**_**y**_^r^*^^, **e**_**y**_^**r**^∗′^^)				
	**x**_1_	**e**_**x**_1__^1**'**^	**e**_**x**_1__^1^	**x**_1_^**a**^	**e**_**x**_1__^**r**^	**e**_**x**_1__^**r'**^	**e**_**y**_^1′^	**e**_**y**_^1^	**y** ^**a**^	**e**_**y**_^**r**^	**e**_**y**_^**r**′^	**e**_**y**_^1^∗′^^	**e**_**y**_^1^*^^	**y** ^a^*^^	**e**_**y**_^r^*^^	**e**_**y**_^**r**^∗′^^
1	**6**	**7**	**6**	**10**	**5**	**7**	6	3	5	2	5	5.4158	2.5665	4.7776	2.2215	4.2473
2	**7**	**6**	**5**	**12**	**4**	**6**	7	5	4	5	7	5.8827	3.1771	6.2226	2.8438	4.9676
3	**8**	**4**	**2**	**15**	**3**	**5**	8	3	9	4	6	6.7973	4.3734	9.0537	4.0632	6.3788
4	**9**	**7**	**5**	**20**	**8**	**10**	7	3	10	2	4	6.4422	3.9089	7.9544	3.5897	5.8308
5	**10**	**8**	**6**	**5**	**2**	**5**	8	5	12	5	7	7.4885	5.2774	11.193	4.9846	7.4451
6	**11**	**10**	**8**	**15**	**5**	**7**	3	2	8	4	6	6.5757	4.0835	8.3678	3.7678	6.0369
7	**12**	**20**	**15**	**25**	**12**	**14**	6	5	7	3	5	6.2393	3.6436	7.3265	3.3193	5.5179
8	**13**	**12**	**7**	**30**	**15**	**18**	8	7	14	6	8	8.3421	6.3937	13.835	6.1226	8.7620
9	**14**	**16**	**12**	**20**	**10**	**15**	11	9	16	10	12	9.7423	8.2250	18.169	7.9893	10.9223
10	**15**	**17**	**13**	**22**	**8**	**12**	8	7	18	5	10	9.0740	7.3509	16.100	7.0982	9.8912

**Table 4:** Intuitionistic fuzzy input-intuitionistic fuzzy output data.

Object *i *	Int. fuzzy input **X** = (**e**_**x**_1__^1**'**^, **e**_**x**_1__^1^, **x**_1_^**a**^, **e**_**x**_1__^**r**^, **e**_**x**_1__^**r'**^)					Int. fuzzy output **y** = (**e**_**y**_^1′^, **e**_**y**_^1^, **y** ^**a**^, **e**_**y**_^**r**^, **e**_**y**_^**r**′^)					Estimated int. fuzzy output **y** ^*^ = (**e**_**y**_^1^∗**'**^^, **e**_**y**_^1^*^^, **y** ^a^*^^, **e**_**y**_^r^*^^, **e**_**y**_^**r**^∗′^^)				
	**e**_**x**_1__^1**'**^	**e**_**x**_1__^1^	**x**_1_^**a**^	**e**_**x**_1__^**r**^	**e**_**x**_1__^**r'**^	**e**_**y**_^1′^	**e**_**y**_^1^	**y** ^**a**^	**e**_**y**_^**r**^	**e**_**y**_^**r**′^	**e**_**y**_^1^∗**'**^^	**e**_**y**_^1^*^^	**y** ^a^*^^	**e**_**y**_^r^*^^	**e**_**y**_^**r**^∗**'**^^
1	**5**	**3**	**4**	**5**	**6**	5	4	12	4	6	5.7505	4.2398	9.9797	4.2398	6.7505
2	**7**	**6**	**7**	**8**	**9**	7	5	7	5	8	5.7737	4.2652	10.045	4.2652	6.7737
3	**5**	**3**	**6**	**8**	**9**	5	3	9	3	6	4.8608	3.2665	7.4714	3.2665	5.8608
4	**4**	**2**	**7**	**9**	**11**	3	1	4	1	4	3.7872	2.0920	4.4446	2.0920	4.7872
5	**3**	**2**	**5**	**7**	**8**	4	2	6	2	5	4.9552	3.3698	7.7377	3.3698	5.9552
6	**6**	**3**	**6**	**7**	**10**	5	4	8	4	6	4.5158	2.8891	6.4987	2.8891	5.5158
7	**5**	**2**	**4**	**9**	**12**	4	3	9	3	5	5.5863	4.0602	9.5168	4.0602	6.5863
8	**6**	**5**	**8**	**13**	**15**	7	5	10	5	8	5.2404	3.6818	8.5417	3.6818	6.2404
9	**8**	**7**	**12**	**15**	**17**	4	3	5	3	5	3.9099	2.2262	4.7905	2.2262	4.9099
10	**15**	**10**	**15**	**20**	**25**	4	2	3	2	5	3.6202	1.9092	3.9736	1.9092	4.6202
